# Exposure to One Antibiotic Leads to Acquisition of Resistance to Another Antibiotic *via* Quorum Sensing Mechanisms

**DOI:** 10.3389/fmicb.2020.580466

**Published:** 2021-01-20

**Authors:** Che-Chi Shu, Wan-Ci Chen, Yao-Duo Chang, Jyy-Ning Chen, Feng-You Liu, Yu-Shan Huang, Chao-Xuan You, En Hsuan Wu

**Affiliations:** Department of Chemical Engineering and Biotechnology, National Taipei University of Technology, Taipei, Taiwan

**Keywords:** dissemination of drug resistantance, selection pressure, nosocomial infection, conjugal genes, *prgX*, *prgQ*, post-antibiotic era, side effect

## Abstract

The vancomycin-resistant Enterococci (VRE) have progressively become a severe medical problem. Although clinics have started to reduce vancomycin prescription, vancomycin resistance has not been contained. We found that the transfer of vancomycin resistance in *Enterococcus faecalis* increased more than 30-fold upon treatment by streptomycin. Notably, treatment with an antibiotic caused the bacteria to become resistant to another. The response was even stronger in the well-studied plasmid pCF10 and the number of transconjugants increased about 100,000-fold. We tested four different antibiotics, and all of them induced conjugal response. Through a mathematical model based on gene regulation, we found a plausible explanation. *Via* quorum sensing, the change of the cell density triggers the conjugation. Moreover, we searched for generality and found a similar strategy in *Bacillus subtilis*. The outcome of the present study suggests that even common antibiotics must not be overused.

## Introduction

The crisis of antibiotic resistance has received considerable attention in recent years (Chatterjee et al., 2011; Brown and Wright, 2016). Much effort has been made to uncover how antibiotic resistance is acquired (Baker et al., 2018). Such efforts have been made to avoid a post-antibiotic era where even common infections can once again lead to death. In the early-to-mid twentieth century, scientists already recognized that treatment with an antibiotic prompted bacteria to develop resistance to it. Moreover, administration of an antibiotic may induce efflux pumps (Papkou et al., 2020), which export antimicrobials and lead to multidrug resistance (Grimsey et al., 2020). To avoid accelerating antibiotic resistance, medical doctors are inclined to use early-discovered antibiotics. The other side effect of administrating antibiotics is that it kills not only the pathogens but also harmless bacteria. The collateral damage on the symbiotic bacteria may cause harm to a patient’s health in the future (Blaser, 2016). Without the contribution of commensal bacteria to intestinal microbiota, patients are more vulnerable to infections (Buffie and Pamer, 2013). Regrettably, there is still another serious side effect of abusing antibiotics, and it has been long overlooked by the medical community. In the present study, we found that administration of an antibiotic reduces the population of vulnerable cells in *Enterococcus faecalis*. Due to quorum sensing, the vulnerable donor cells spread another antibiotic resistance.

**Figure 1:**
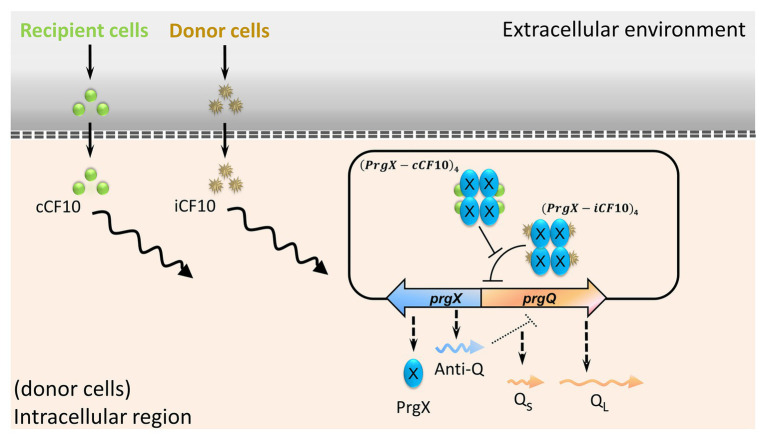
The main regulation of conjugal genes in plasmid pCF10. Peptides cCF10 and iCF10, through PrgX protein, determine the expression level of *prgQ*. The induction of *prgQ* results in the production of Q_L_ RNA, which triggers the conjugation.

Enterococci are multiple drug-resistant nosocomial bacteria (Tendolkar et al., 2003). The opportunistic pathogen *E. faecalis* causes urinary tract infections and endocarditis (Paulsen et al., 2003). Typically, infections are present in patients who have an impaired immune system and who have received multiple courses of antibiotics (O’Driscoll and Crank, 2015). Shortly after the first isolation of vancomycin-resistant Enterococci (VRE) in 1988, they spread with unanticipated rapidity (Cetinkaya et al., 2000). Enterococci are proficient in the dissemination of genes that encode drug resistance (Arias and Murray, 2009). In *E. faecalis*, pheromone-inducible conjugation is a highly efficient process of transferring antibiotic resistance and virulence (Hirt et al., 2018). Plasmid pCF10 is one of the most well-studied conjugal systems (Hirt et al., 2005). The regulation of conjugal genes relies on the ratio of cCF10 to iCF10 (Chatterjee et al., 2013). The mating pheromone cCF10 is a heptapeptide (LVTLVFV). An inhibitor or a self-sensing signal iCF10 is another heptapeptide (AITLIFI). Both iCF10 and cCF10 are quorum-sensing signals. In Figure 1, the left shows that cCF10 and iCF10 are released by recipient and donor cells (Nakayama et al., 1994; Dunny, 2007), respectively. The donor cells uptake these two peptides through active transporters (Leonard et al., 1996). After importation, the peptides compete for PrgX. This protein is either bound with iCF10 to form (PrgX-iCF10)_4_ or bound with cCF10 to form (PrgX-cCF10)_4_ (Erickson et al., 2019). The ratio of donor to recipient cells determines the ratio of (PrgX-iCF10)_4_ to (PrgX-cCF10)_4_. The *prgX* and *prgQ* are the main regulatory genes of conjugal response (Shu et al., 2011). The *prgQ* and its downstream genes are in charge of conjugation, and the complex of PrgX protein regulates its expression. The (PrgX-iCF10)_4_ represses the transcription initiation of *prgQ* by blocking the binding site of RNA polymerase but (PrgX-cCF10)_4_ does not (Chen et al., 2017). In addition to the PrgX protein, the *prgX* gene utilizes sense-antisense interaction to repress the expression of *prgQ* because these two genes are in opposite directions with 223 nt overlapped (Chatterjee et al., 2011). The transcription of *prgX* produces truncated RNA named as Anti-Q, which terminates the transcription of *prgQ* and causes it to form Qs RNA (Shokeen et al., 2010). If the transcription of *prgQ* is not terminated by Anti-Q, it produces a longer RNA Q_L_. Only when cells are induced by cCF10, the gene *prgQ* abundantly produces QL RNA (Bensing and Dunny, 1997). Both Qs and Q_L_ encode iCF10, but only Q_L_ triggers the expression of downstream conjugal genes (Chatterjee et al., 2011; Erickson et al., 2020). The hospital isolated vancomycin-resistant plasmid, pMG2200, also has *prgQ* and *prgX*, which regulates its conjugation. Namely, it utilizes the same regulatory strategy as pCF10 (Zheng et al., 2009). In the present study, we aim to discover how such conjugations respond to administration of an antibiotic.

## Materials and Methods

### The Conjugation With the Presence of Antibiotics

**Figure 2:**
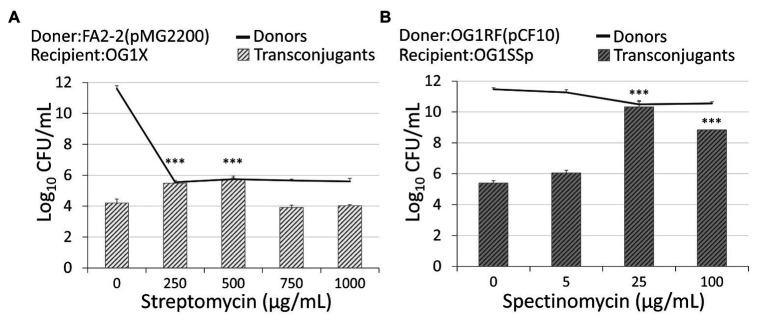
Administration of antibiotics affects the conjugation rate. **(A)** An administration of streptomycin of 250 *μ*g/ml or 500 μg/ml increased the conjugation of plasmid pMG2200 encoding vancomycin resistance. **(B)** An administration of spectinomycin of 25 μg/ml or 100 μg/ml leads to an increase in the conjugation of plasmid pCF10 (***indicates *p* < 0.001, in comparison to the case without an antibiotic).

For the conjugation of pCF10 shown in Figure 2, the recipients OG1SSp are resistant to 250 μg/ml spectinomycin and 1,000 μg/ml streptomycin (Hirt et al., 2005). The donors OG1RF (Torres et al., 1991) are resistant to 200 μg/ml rifampicin, and the plasmid pCF10 is resistant to 10 μg/ml tetracycline. Cells were cultured overnight in Todd-Hewitt Broth (THB) at 37°C. We then washed the cells twice and made a 1:10 dilution. Next, we treated separately donor cells and recipient cells with the spectinomycin concentration of 0, 250, 500, 750, or 1,000 μg/ml. After 60 min of incubation at 37°C, one volume of recipients was mixed with 10 volume of donors. The cells were continuously exposed to spectinomycin, and the mating lasted for 3 h. Serial dilutions of liquid samples were plated on agar. All data were collected from at least three bio-replicates; they are from different colonies while preparing overnight culture. While comparing two sets of data, we applied the permutation test (Fraker and Peacor, 2008) to obtain the *p*-value. The permutation test, from the original data sets, randomly generates replicate data and then calculates the *F*-value. The probability of any permutation data that produces an F-ratio more extreme than that of the actual data is the *p*-value.

For the conjugation of pMG2200 (Zheng et al., 2009) shown in Figure 2, the recipients OG1X (Ike et al., 1983; Flannagan et al., 2003) are resistant to 1,000 μg/ml streptomycin. The donors FA2-2 (Jacob and Hobbs, 1974; Flannagan et al., 2003) are resistant to 25 μg/ml rifampicin and the plasmid pMG2200 resistant to 10 μg/ml vancomycin. We cultured cells in THB at 37°C. We treated separately donors and recipients with the streptomycin concentration of 0, 5, 25, or 100 μg/ml. We then incubated cells overnight. We washed cells and took one volume of recipients mixed with five volume of donors. After 3 h of liquid mating, serial dilutions of samples were plated on agar. We prepared the plates of transconjugant cells with 1,000 μg/ml streptomycin and 10 μg/ml vancomycin. Note that we conducted negative control, and both pure donor and pure recipient cells were found with no colony on the plates. We also conducted such negative controls with proper antibiotics in other tests of conjugation to exclude the possibility of a spontaneous mutation.

### The β-Galactosidase Assay of Plasmid pBK2 With the Treatment of Antibiotics

**Figure 3:**
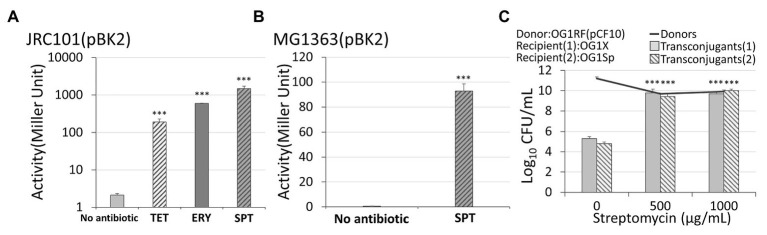
The response of conjugal genes to different types of antibiotics. **(A)** In *Enterococcus faecalis* with plasmid pBK2, administration of 5 μg/ml spectinomycin, 5 μg/ml tetracycline, or 0.03 μg/ml erythromycin caused the conjugal response. **(B)** In *Lactococcus lactis* with plasmid pBK2, administration of spectinomycin results in the conjugal response. **(C)** An administration of streptomycin on the vulnerable donor cells OG1RF, resistant recipients OG1X, and vulnerable recipients OG1Sp increased the conjugation frequency. The transconjugant (1) and (2) are OG1X(pCF10) and OG1Sp(pCF10), respectively (***indicates p < 0.001, in comparison to the case without an antibiotic).

For the β-galactosidase assay shown in Figure 3, we use JRC101 instead of wild-type OG1RF. The difference between these two strains is that JRC101 (Chandler et al., 2005b) has a mutation of *ccfA* gene so it produces no cCF10. The reasons for using JCR101 are as follows. In nature, OG1RF(pCF10) produces little cCF10 because the PrgY protein encoded in the plasmid prevents cells from releasing cCF10 (Chandler et al., 2005a). However, plasmid pBK2 has no *prgY* gene (Shokeen et al., 2010). The gene map of pBK2 is in Supplementary Figure S1. Consequently, OG1RF(pBK2) produces the same amount of cCF10 as recipient cells. The extra cCF10 may severely change the conjugal response. To avoid it, we used JRC101 with plasmid pBK2. For the experiment with *Lactococcus lactis*, we transformed the pBK2 into strain MG1363 (Linares et al., 2010). *Enterococcus faecalis* and *L. lactis* were incubated overnight in THB at 37°C and in M17 at 32°C with the treatment of 5 and 30 μg/ml spectinomycin, respectively. Then, we induced cells with 0.5 μg/ml cCF10. After 90 min of incubation, we placed tubes on ice for 5 min and added 1/5 volume of toluene. The following β-galactosidase assays were performed as detailed by Miller (1972).

**Figure 4:**
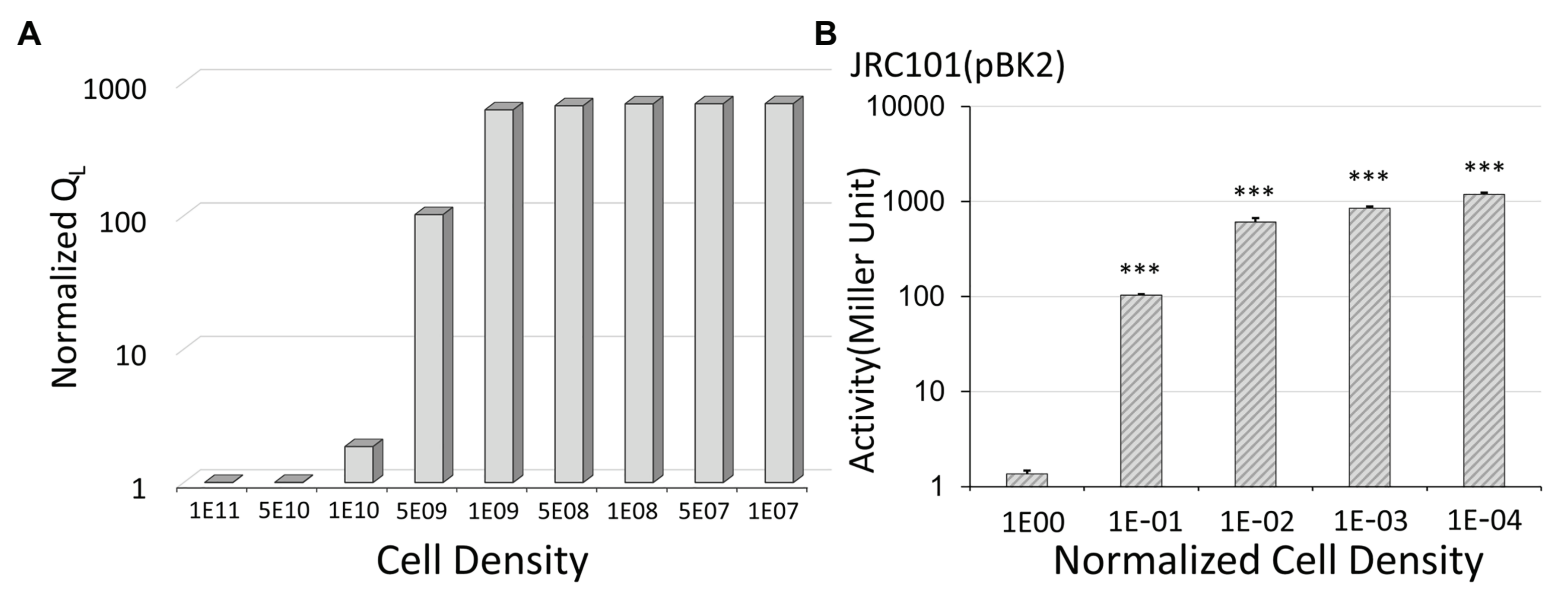
The effect of cell density on conjugal response. **(A)** The simulation results show that low donor density leads to a high expression level. Note that we use the inverted scale of the x-axis. For the y-axis, the normalized Q_L_ is the expression levels divided by the lowest one obtained at a cell density of 10^11^ CFU/ml. **(B)** In *Enterococcus faecalis* with plasmid pBK2, low cell density results in the high expression of the conjugal gene. For the x-axis, the normalized cell density is the cell density divided by the overnight cell density (***indicates p < 0.001, in comparison to the case with a normalized cell density of one).

The steps for the assay of plasmid pBK2 with different cell densities (Figure 4) are as follows. After culturing JRC101(pBK2) overnight in THB at 37°C, we made a 1:10, 1:100, 1:1,000 or 1:10,000 dilutions. The normalized cell density is the cell density divided by the density of the overnight culture. After 2 h of incubation, we then induced cells with 30 ng/ml cCF10 for 90 min followed by β-galactosidase assay.

### The Liquid Mating With Two Different Recipient Cells

For the liquid mating in Figure 3C, we use OG1RF (resistant to 200 μg/ml rifampicin) as donor cells harboring plasmid pCF10 (resistant to 10 μg/ml tetracycline). Two recipient cells are OG1Sp (Kristich et al., 2007; resistant to 1,000 μg/ml spectinomycin) and OG1X (resistant to 1,000 μg/ml streptomycin). After overnight culture in THB at 37°C, we washed cells twice and made a 1:10 dilution. We then separately treated donor cells and recipient cells with the streptomycin concentration of 0, 500, or 1,000 μg/ml. After 60 min of incubation at 37°C, we mixed one volume of each recipient with 10 volume of donors. We conducted liquid mating for 3 h. Serial dilutions of liquid samples were plated on agar.

### The Mathematical Model

A mathematical model incorporating gene regulation of conjugal response in Figure 1 is in the literature (Chatterjee et al., 2013). In the present study, we use exactly the same equations. We also apply exactly the same parameter values to the model, except for the volume conversion factor and the secretion rate constants. The volume conversion factor describes the volume of *E. faecalis* in the broth. We realized that the volume of *E. faecalis* is smaller than the volume that has been assigned in the original model. Due to the change of cellular volume, we modified the secretion rate constants. The Supplementary Tables S1-S3 listed equations, nomenclatures, and parameter values. Briefly, the equations start with a variable describing the DNA configuration of *prgQ*-*prgX* region in donor cells. If DNA is bound with (PrgX-iCF10)_4_, the transcription rate constant of *prgQ* is low. Oppositely, if cells are dominated by (PrgX-cCF10)_4_, *prgQ* is highly expressed. The configuration of DNA also affects the transcription rate constant of *prgX*, which produces PrgX protein and truncated RNA in *prgX* direction, Anti-Q. Note that Anti-Q and the mRNA of PrgX are in different regions. The mRNA of PrgX is not under the effect of the truncated RNA in *prgQ* direction but Anti-Q is. To determine the concentration of Anti-Q, the model has to calculate the amount of the truncated RNA in *prgQ* direction and account for its influence on Anti-Q. In model, Anti-Q interferes in the transcription of *prgQ* to separate Qs from Q_L_. Both Qs and Q_L_ produce iCF10. Note that recipient cells produce cCF10. The iCF10 and cCF10 in donor cells compete for PrgX protein to form (PrgX-iCF10)_4_ and (PrgX-cCF10)_4_, respectively. Thereby, the concentration of iCF10 and cCF10 controls the configuration of the DNA. The generation of conjugation protein is then calculated from the concentration of Q_L_. In the present study, we use Q_L_ to indicate the conjugal level.

### The Liquid Mating With Different Ratio of Donor to Recipient Cells

For conjugation of plasmid pCF10, we used OG1RF (Torres et al., 1991) and OG1SSp (Torres et al., 1991) as donor and recipient cells, respectively. After culturing overnight in THB at 37°C, we washed cells twice and made a 1:10 and a 1:100 dilution for high and low donor density, respectively. We treated recipient cells with the same procedure as that of high donor density. After 1-h incubation, we mixed one volume of recipients with 10 volume of donors and allowed the mating to last for 3 h. Serial dilutions of liquid samples were plated on agar. For the conjugation of plasmid pMG2200, we used FA2-2 and OG1X as donor and recipient cells, respectively. It followed the same procedure except that we made a 1:10^7^ dilution for low donor density.

### The Information of Strains and Plasmids

From the lab of Prof. Gary M. Dunny in the University of Minnesota, we obtained *E. faecalis* strains OG1RF, JRC101, OG1SSp, OG1Sp, and OG1X and plasmids pCF10 and pBK2. The *E. faecalis* strain FA2-2 and the plasmid pMG2200 are from the lab of Prof. Haruyoshi Tomita in the Gunma University. The *L. lactis* strain MG1363 is from the lab of Prof. Cheng-Kang Lee in National Taiwan University of Science and Technology.

## Results

### Dissemination of Vancomycin Resistance

In rifampicin-resistant donor cells, FA2-2, conjugal plasmid pMG2200 (Zheng et al., 2009) encodes vancomycin resistance. Through conjugation in liquid mating, streptomycin-resistant recipients OG1X acquired pMG2200 and became vancomycin-resistant transconjugants. The methods and strain information are detailed in section Materials and Methods. Without administration of streptomycin in donor cells, 1.6 × 10^4^ colony forming units (CFUs) per milliliter of transconjugants were identified (first left bar in Figure 2A). While maintaining the environmental concentration of streptomycin at 250 μg/ml, the conjugation increased significantly. Specifically, when the concentration of streptomycin reached 500 μg/ml, the number of transconjugants increased to 5.28 × 10^5^ CFU/ml (Figure 2A). Compared with the case that did not receive treatment with streptomycin, the number of transconjugants increased more than 30-fold. The donor density decreased from 4.02 × 10^11^ to 4.63 × 10^5^ CFU/ml (Figure 2A, black line). In other words, the conjugation rate per donor increased by more than 10 million times. This result is not from the spontaneous mutation of donor cells becoming streptomycin resistance because we treated donor cells with streptomycin without recipients, and we found no colony on the plate of transconjugants. Figure 2A suggests that the administration of streptomycin promoted the conjugal response of vancomycin-resistant plasmids, which increased the number of VRE. When further increasing the concentration of streptomycin, it is highly likely that vulnerable donors lose vitality, causing the number of transconjugants to decrease.

### Conjugal Response of pCF10

The plasmid pMG2200 contains part of the pheromone-responsive genes *prgX* and *prgQ* identical to those in the plasmid pCF10 (Hirt et al., 2005; Zheng et al., 2009). If these genes are responsible for the induction of conjugation during treatment with antibiotics, the plasmid pCF10 should behave similarly to pMG2200. We allowed rifampicin-resistant donors, OG1RF, to transfer plasmid pCF10 (resistant to tetracycline) to the recipients (spectinomycin‐ and streptomycin-resistant OG1SSp). Figure 2B shows that the treatment with spectinomycin over 4 h considerably increased the conjugation frequency. Interestingly, not only the streptomycin but also spectinomycin stimulated the transfer of plasmids. When the concentration of spectinomycin was raised from 0 to 25 μg/ml, the number of transconjugants increased significantly from 2.52 × 10^5^ to 2.11 × 10^10^ CFU/ml. The number of transconjugants increased about 100,000-fold. When we further increased the concentration of spectinomycin to 100 μg/ml, the number of transconjugants decreased to 7 × 10^8^ CFU/ml. The trend is similar to that for pMG2200. Next, we examined the influence of a longer treatment with antibiotics. Compared with the case receiving no treatment, cells treated with antibiotics achieved a much higher conjugation frequency (2.85 × 10^9^ CFU/ml of transconjugants) after exposure to 25 μg/ml spectinomycin for up to 20 h (Supplementary Text S1 and Supplementary Figure S2).

### Main Regulatory Genes *prgX* and *prgQ*

To confirm that genes *prgX* and *prgQ* are responsible for the conjugal behaviors in Figure 2, we used an engineered plasmid, pBK2. In pBK2 (Supplementary Figure S1), the only genes from the plasmid pCF10 are *prgX* and *prgQ*. The backbone of pBK2 is pCI3340 (Hayes et al., 1990), a plasmid in *L. lactis*. In pCF10, Q_L_ RNA leads to the expression of downstream conjugal genes, and thus it serves as an indicator of conjugation (Chatterjee et al., 2013). In pBK2, a reporter *lacZ* gene replaced the part of conjugal gene *prgQ* that encodes Q_L_ RNA (Breuer et al., 2017). Thereafter, we measured the beta-galactosidase activity to quantify the level of conjugation (Shokeen et al., 2010). In this simplified system, cells harboring pBK2 are imitators of donor cells. As for recipient cells, they secreted pheromone cCF10, so we added synthetic peptide cCF10 to mimic them.

When exposed to 0.5 μg/ml of the pheromone cCF10, plasmid pBK2 was uninduced if no antibiotics were administered (the left first column of Figure 3A). When the cells were treated with 5 μg/ml of spectinomycin from overnight culture, the beta-galactosidase activity increased significantly (the right first column of Figure 3A). This result is consistent with the conjugal behaviors of the plasmid pCF10 (Figure 2B). Note that the expression level may be a little sensitive to the experimental conditions, but the treatment with antibiotics led to a great induction of cells (Supplementary Text S2 and Supplementary Figure S3). To further exclude the influence from other genes in *E. faecalis*, we transformed plasmid pBK2 into *L. lactis*, MG1363. Remarkably, the treatment with 30 μg/ml of spectinomycin significantly increased the expression level of *prgQ*, even in *L. lactis* (Figure 3B). This result suggests that genes *prgX* and *prgQ* are responsible for the induction of the conjugal response by antibiotics.

### Treatment With Different Antibiotics

The antibiotics shown in Figures 2A,B are streptomycin and spectinomycin, respectively. The results imply that the induction of the conjugal response is not restricted to only one type of antibiotic. To confirm this, we treated cells harboring pBK2 with 5 μg/ml of tetracycline, and cells were induced (Figure 3A). Similarly, we treated cells with 5 μg/ml of erythromycin, but it is too much. We then reduced the concentration of erythromycin to 0.03 μg/ml and cells were induced (Figure 3A). The result suggests that the conjugal genes in donor cells can be induced by various antibiotics. To understand whether the vulnerable recipient cells might compromise the conjugation, we used OG1RF (pCF10) as donor cells and two recipient cells are OG1X and OG1Sp, which are resistant to streptomycin and spectinomycin, respectively. We then administered different concentrations of streptomycin (Figure 3C). Remarkably, streptomycin induced the conjugal response in donor cells without hindering the vulnerable recipient cells from acquiring plasmids. The conjugation increased more than 10,000-fold even for the recipient OG1Sp, which is sensitive to streptomycin. This outcome suggests that the type of antibiotic administered may not be important. Treatment with an antibiotic helps *E. faecalis* to become resistance to another antibiotic.

### Insights From the Mathematical Model

We applied a mathematical model to describe the conjugal regulation of genes *prgX* and *prgQ* and used the amount of Q_L_ to quantify the expression level of the conjugal gene (Chatterjee et al., 2013). The results are shown in Figure 4A. In the y-axis, we use normalized Q_L_, which are the expression levels divided by the lowest one. We obtained the lowest value of Q_L_ at a cell density of 10^11^ CFU/ml. Note that we use the inverted scale of the x-axis in Figure 4. The simulation results indicate that the expression level increased when donor density decreased. To confirm it, we examined the influence of cell density on the beta-galactosidase activity in pBK2 (Figure 4B). The normalized cell density is the cell density divided by the overnight cell density. The outcome of the mathematical model (Figure 4A) is consistent with experimental observations (Figure 4B). From the results of the mathematical model and the expression level of the conjugal response in pBK2, we realized that low donor density stimulates the expression of the conjugal gene, thus facilitating the dissemination of plasmids. This is because donor cells release a quorum-sensing signal, iCF10, to suppress the conjugal response. Treatment with an antibiotic lowered cell density and thereby prompted the transfer of the plasmid that encodes resistance to another antibiotic.

### Influence of Donor Density

**Figure 5:**
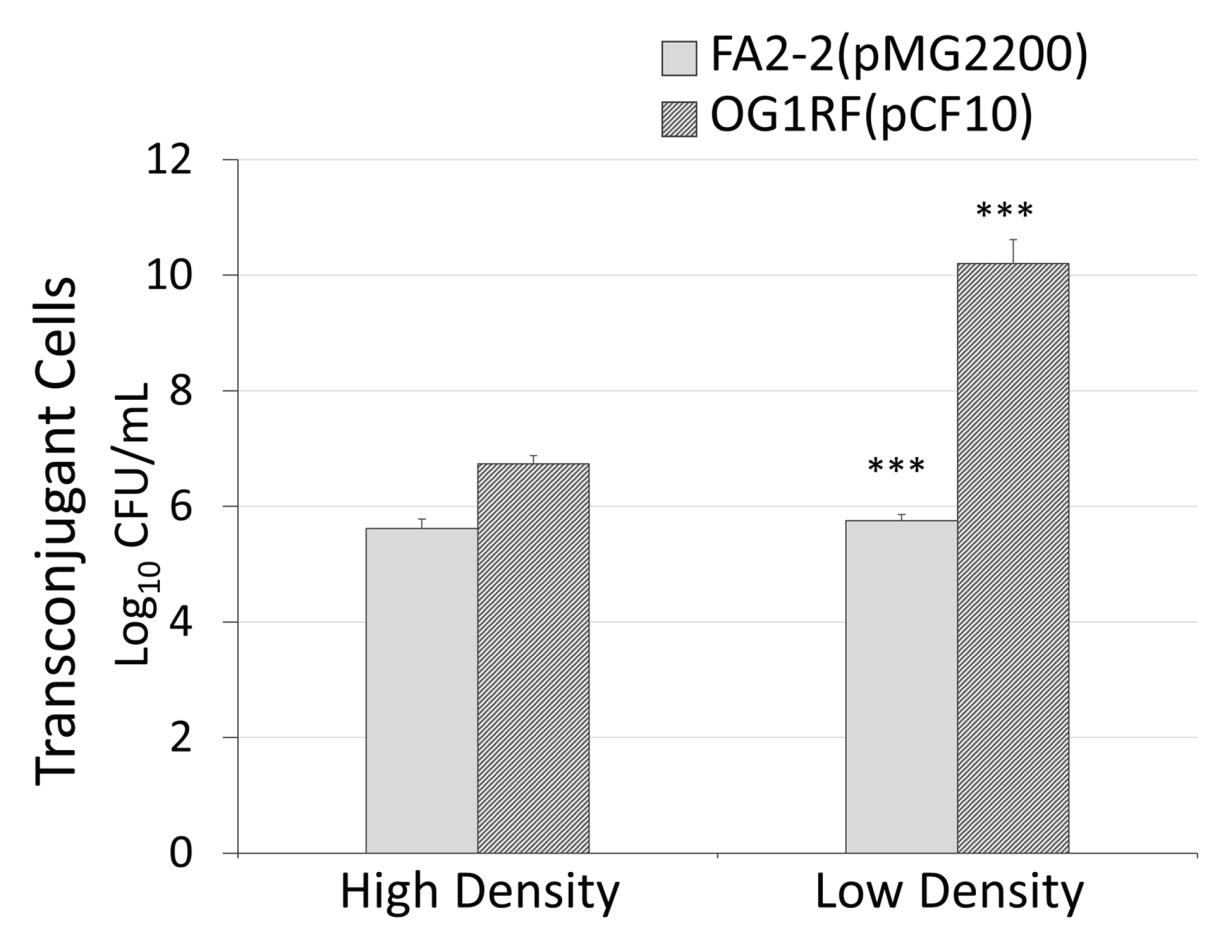
Low donor density leads to a high conjugation rate. In x-axis, high density is a 1:10 dilution to the overnight donor culture and low density are 1:10,000,000 and 1:100 to an overnight donor culture of FA2-2 (pMG2200) and OG1RF (pCF10), respectively (***indicates p < 0.001, in comparison to the case with high cell density).

To further confirm that donor density is the cause of antibiotic-induced conjugal response, we examined the influence of donor density on conjugation. We conducted liquid mating by mixing the same amount of recipient cells with either a high or low density of donor cells. We did different dilutions to overnight culture. For FA2-2(pMG2200), the dilutions of donor cells were 1:10 and 1:10,000,000. For OG1RF(pCF10), the dilutions of overnight culture were 1:10 and 1:100. We chose these values based on the donor cell density illustrated in Figure 2. In Figure 5, the gray and black columns display the number of transconjugant cells for the plasmids pMG2200 and pCF10, respectively. As expected, systems with low donor density appeared to have a high conjugation frequency. This outcome is consistent with Figure 2, where transconjugant cells increase with a decrease in donor density (lines in Figure 2), as long as the concentration of the antibiotic is not too high.

## Discussion

In addition to pMG2200, many other plasmids have the same regulatory genes. From National Center for Biotechnology Information (NCBI) blast results, plasmids pE512, EF62pB, pN48037F-2, and Efsorialis-p2, have identical sequences (Supplementary Table S4) as *prgX* and *prgQ* in pCF10. The conclusion of this study might also apply to these plasmids. Chatterjee et al. (2013) revealed that the plasmid pCF10 is capable of sensing its own density through the quorum sensing signal iCF10 encoded in *prgQ* and thus the conjugation frequency decreases at a high donor density. In the present study, we discovered that the administration of antibiotics lowers donor density but failed to stop the plasmid spread. Thus, cells treated with an antibiotic were induced to spread resistance to other types of antibiotics. Other plasmids in *E. faecalis* may also have a similar regulatory pattern as that of pCF10. Both plasmids pAD1 and pAM373 showed a decrease of conjugation frequency at a high donor density (Bandyopadhyay et al., 2016). Plasmids pMG2201 (Zheng et al., 2009) and pTEF1 (Paulsen et al., 2003) have been reported to have the same self-sensing signal and the same conjugal response as pAD1. All these plasmids might have the same response to administration of antibiotics. Recently, the dissemination of linezolid resistance through the plasmids with conjugal systems similar to that of pCF10 and pAD1 has also been found (Zou et al., 2020). Enterococci is notorious for nosocomial infection (Bereket et al., 2012), and the present study indicated that administration of a common antibiotic led to the incremental dissemination of plasmids encoding resistance to other antibiotics, including vancomycin. Thereby, it is better to stop overusing antibiotics, even the common ones! Note that not only the donor cells of *E. faecalis* are capable of sensing their own density through a quorum-sensing signal, but also the donors of *Bacillus subtilis* (Itaya et al., 2006) release a quorum-sensing signal phr*_LS20_ to inactive the conjugation (Singh et al., 2013). Such a quorum-sensing strategy is not unique in *E. faecalis*.

## Data Availability Statement

The original contributions presented in the study are included in the article/Supplementary Material, further inquiries can be directed to the corresponding author.

## Author Contributions

C-CS initiated the idea, designed the experiment, and formulated the model. W-CC, Y-DC, and J-NC conducted the experiment. Y-SH repeated the experiment. F-YL and C-XY performed the simulation and analyzed the data. C-CS and EHW wrote the manuscript. All authors contributed to the article and approved the submitted version.

### Conflict of Interest

The authors declare that the research was conducted in the absence of any commercial or financial relationships that could be construed as a potential conflict of interest.
