# Supplementary material for: Exposure to One Antibiotic Leads to Acquisition of Resistance to Another Antibiotic via Quorum Sensing Mechanisms
Source: Front Microbiol. 2021 Jan 20;11:580466. doi: 10.3389/fmicb.2020.580466 (PMC7855173; doi:10.3389/fmicb.2020.580466)
Supplement: Supplementary file 1 [file Data_Sheet_1.pdf]

Supplementary Material

1 Text S1 The conjugation with a long-time treatment of spectinomycin

For the conjugation of pCF10 with the treatment of spectinomycin for 20 hours, we used OG1RF and OG1SSp as donor and recipient cells, respectively. We separately cultured overnight donor cells and recipient cells in Todd-Hewitt Broth (THB) at 37°C with the treatment of 25 μg/ml spectinomycin. We then washed cells twice and made a 1:10 dilution. We maintained the concentration of spectinomycin at 25 μg/ml and incubated cells for 60 min. Then, we mixed one volume of recipients with ten volume of donors in liquid mating for 3 hours. Serial dilutions of liquid samples were plated on agar. The results are in Fig. S2. The exposures to antibiotics notably increased the conjugation.

2 Text S2 The response of pBK2 with a short-time treatment of streptomycin

In Fig. 3a, the overnight culture of cells was treated by antibiotics. Here, we examined the cellular behaviors under a short-time treatment with spectinomycin. We did a 1:10 dilution to the overnight culture of JRC101(pBK2) and then administrated 5 μg/ml spectinomycin. Cells were exposed to spectinomycin for 2 hours incubation followed by 1.5 hours of induction by 30 ng/ml cCF10. The results are in Fig. S3. Administration spectinomycin increased beta-galactosidase activity.

3 Figures and Tables

3.1 Supplementary Figures

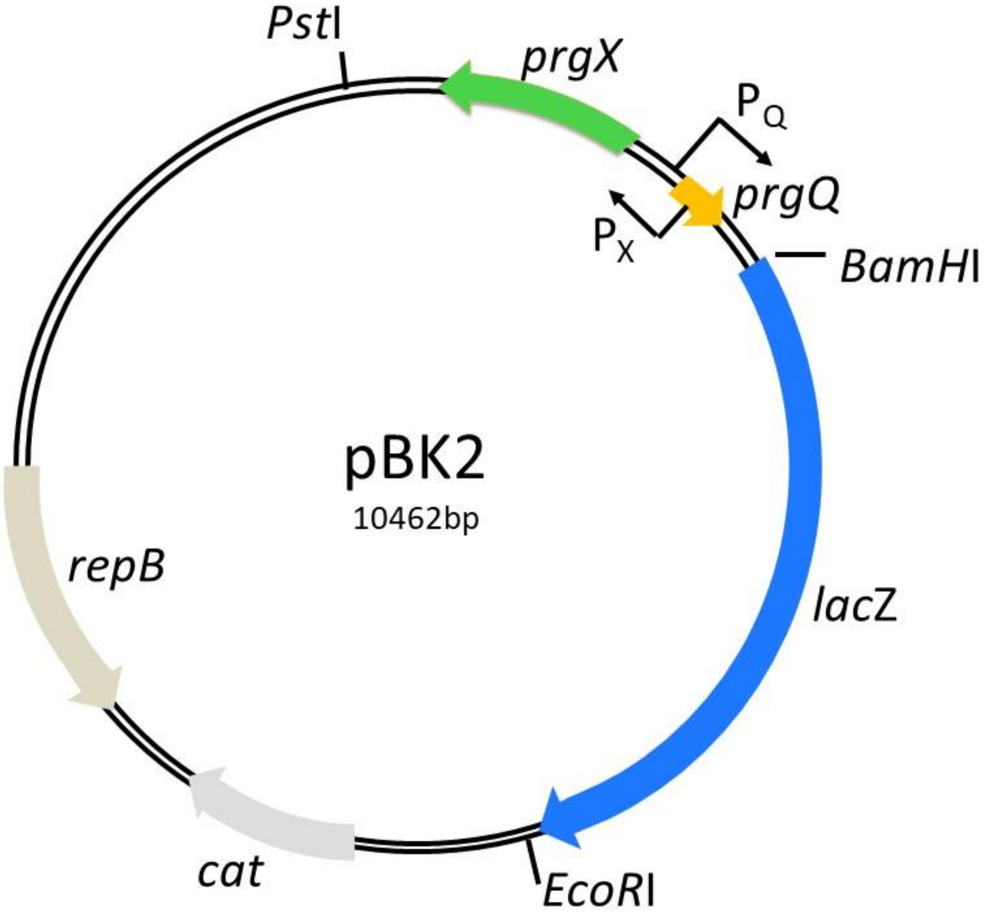

Supplementary Figure S1. The gene map of pBK2.
The backbone of pBK2 is pCI3340, a plasmid in *Lactococcus lactis*. Genes *prgX* and *prgQ* are from the plasmid pCF10. The codon of gene *prgQ* encoding Q_L RNA, which is the conjugal determinant, was replaced by the *lacZ* in pBK2.

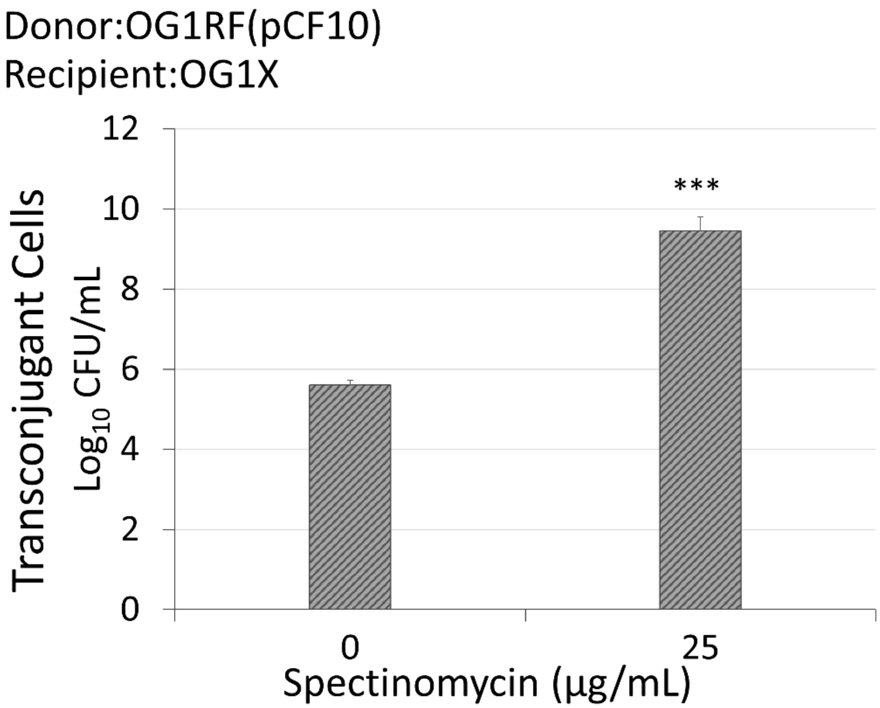

Supplementary Figure S2. The conjugation of pCF10 with a long-time treatment of spectinomycin.

In comparison to that of no treatment, administration of 25 µg/ml spectinomycin increases the number of transconjugant cells. (*** indicates p-value <0.001)

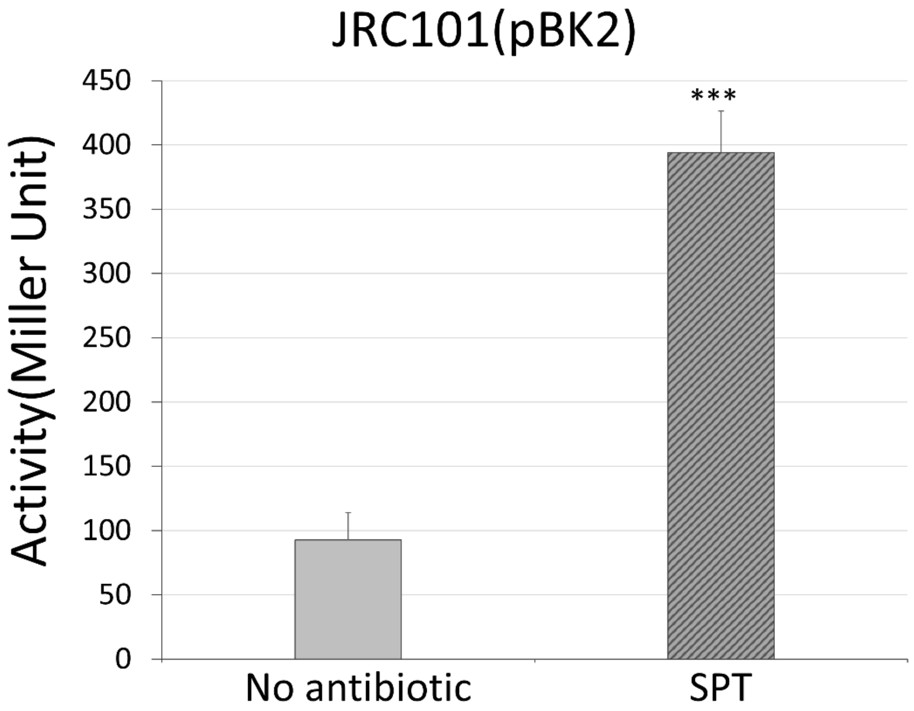

Supplementary Figure S3. The response of the conjugal gene to a short-time treatment of streptomycin.
A 3.5-hour treatment with streptomycin increases beta-galactosidase activity. (*** indicates p-value <0.001)

3.2 Supplementary Tables

Table S1 : Equations of mathematical model

$$[O]=\frac{[N]\cdot[I]^4}{[I]^4+K_{DNA}\cdot[C]^4}$$

$$\frac{d[Q_S]}{dt}=\left(k_{P_Q,loop}[O]+k_{P_Q,unloop}([N]-[O])\right)\left(\frac{K_{Q-X_t}\cdot[X_t]}{1+K_{Q-X_t}\cdot[X_t]}\right)-(\lambda_{Q_S}+\mu_D)[Q_s]$$

$$\frac{d[Q_L]}{dt}=\left(k_{P_Q,loop}[O]+k_{P_Q,unloop}([N]-[O])\right)\left(\frac{1}{1+K_{Q-X_t}\cdot[X_t]}\right)-(\lambda_{Q_L}+\mu_D)\left[Q_L\right]$$

$$\frac{d[Q_t]}{dt}=[k_{Q_t,loop}[O]+k_{Q_t,unloop}([N]-[O])]-k_t[X]\cdot[Q_t]-k_t[X_t][Q_t]-(\lambda_{Q_t}+\mu_D)[Q_t]$$

$$\frac{d[X]}{dt}=k_{P_X,loop}[O]+k_{P_X,unloop}([N]-[O])-k_t[X]\cdot[Q_t]-(\lambda_X+\mu_\mathrm{D})[X]$$

$$\frac{d[X_t]}{dt}=k_{X_t,loop}[O]+k_{X_t,unloop}([N]-[O])-(k_{P_Q,loop}[O]+k_{P_Q,unloop}([N]-[O]))\left(\frac{K_{Q-X_t}\cdot[X_t]}{1+K_{Q-X_t}\cdot[X_t]}\right)$$
$$-k_t[X_t][Q_t]-(\lambda_{X_t}+\mu_D)[X_t]$$

$$\frac{d[i]}{dt}=k_i([Q_s]+[Q_L])\phi\left[D+T\right]-k_{T_i}\cdot([i]-[I])\phi\left[D+T\right]-\lambda_i\cdot[i]$$

$$\frac{d[I]}{dt}=k_{T_i}([i]-[I])-(\lambda_I+\mu_D)[I]$$

$$\frac{d[c]}{dt}=k_c\phi\left[R\right]-k_{T_c}([c]-[C])\phi\left[D+T\right]-\lambda_c[c]$$

$$\frac{d[C]}{dt}=k_{T_c}([c]-[C])-(\lambda_C+\mu_D)[C]$$

$$\frac{d[Prg]}{dt}=k_{Prg}\left[Q_L\right]-(\lambda_{Prg}+\mu_D)\left[Prg\right]$$

$$\frac{d[D]}{dt}=\mu_D[D]$$

$$\frac{d[R]}{dt}=\mu_R[R]-k_{conju}\left([D]+[T]\right)\cdot\left(\frac{[R]}{[D]+[T]+[R]}\right)\cdot[Prg]$$

$$\frac{d[T]}{dt}=\mu_D[T]+k_{conju}\left([D]+[T]\right)\cdot\left(\frac{[R]}{[D]+[T]+[R]}\right)\cdot[Prg]$$

Table S2: List of variables

variable	Description
O	The ratio of the DNA bound by (PrgX-iCF10)$_4$
Q_S	Q_S RNA
Q_L	Q_L RNA
Q_t	truncated RNA in prgQ direction
X	mRNA producing PrgX
X_t	Anti-Q RNA
i	extracellular iCF10
I	intracellular iCF10
c	extracellular cCF10
C	intracellular cCF10
Prg	conjugation proteins
D	donor cells
R	recipient cells
T	transconjugant cells

Table S3: List of parameters values

Parameter	Description	Value
N	Plasmid copy number	5
μ_D	Specific growth rate of donor/transconjugant cells	2.58×10^{-4} s^{-1}
μ_R	Specific growth rate of recipient cells	3.35×10^{-4} s^{-1}
$k_{P_Q,loop}$	Transcription rate of full-length P_Q RNA in looped state	7.23×10^{-3} s^{-1}
$k_{P_Q,unloop}$	Transcription rate of full-length P_Q RNA in unlooped state	8.87×10^{-2} s^{-1}
$K_{X_t,loop}$	Transcription rate of Xt in looped state	1.02×10^{-2} s^{-1}
$k_{X_t,unloop}$	Transcription rate of Xt in unlooped state	1.2×10^{-3} s^{-1}
$k_{P_X,loop}$	Transcription rate of X in looped state	8.23×10^{-3} s^{-1}
$k_{P_X,unloop}$	Transcription rate of X in unlooped state	1.21×10^{-2} s^{-1}
$k_{Q_t,loop}$	Transcription rate of Q_t in looped state	1.80×10^{-3} s^{-1}
$k_{Q_t,unloop}$	Transcription rate of Q_t in unlooped state	1.08×10^{-2} s^{-1}
k_{conju}	Conjugation rate constant	2×10^{-4}(nM)$^{-1}$ s^{-1}
k_i	Generation rate of extracellular iCF10	0.5 s^{-1}
ϕ	Volume conversion factor	1×10^{-13} s^{-1}
k_{T_i}	Transport rate constant of iCF10	1×10^{-4} s^{-1}
k_{T_C}	Transport rate constant of cCF10	1×10^{-3} s^{-1}
k_c	Generation rate of extracellular cCF10	0.6 s^{-1}
k_t	Rate constant of interaction between X RNA and Q_t RNA	1×10^{-3}(nM)$^{-1}$ s^{-1}
K_{Q-X_t}	Equilibrium constant of Q and X_t interaction	4.43(nM)$^{-1}$
K_{DNA}	Ratio of equilibrium binding constant of cCF10 to PrgX and iCF10 to PrgX	1×10^{6} s^{-1}
k_{Prg}	Generation rate of membrane protein PrgB	1×10^{-3} s^{-1}
λ_{Q_S}	Degradation rate of Q_S RNA	1×10^{-4} s^{-1}
λ_{Q_L}	Degradation rate of Q_L RNA	1×10^{-1} s^{-1}
λ_{X_t}	Degradation rate of X_t RNA	3.851×10^{-4} s^{-1}
λ_{Q_t}	Degradation rate of Q_t RNA	1×10^{-3} s^{-1}
λ_X	Degradation rate of X RNA	1×10^{-4} s^{-1}
λ_I	Degradation rate of intracellular iCF10	1×10^{-6} s^{-1}
λ_C	Degradation rate of intracellular cCF10	1×10^{-6} s^{-1}
λ_i	Degradation rate of extracellular iCF10	1×10^{-6} s^{-1}
λ_c	Degradation rate of extracellular cCF10	1×10^{-6} s^{-1}
λ_{Prg}	Degradation rate of PrgB membrane protein	1×10^{-3} s^{-1}

Table S4: The blast result of *prgX* and *prgQ* genes (pCF10 bases 7029 to 8263)

Description	***Query cover (%)***	***E value***	***Per. Identities (%)***
Enterococcus faecalis plasmid pCF10	100	0	100.00
Enterococcus faecalis plasmid pMG2200	100	0	99.92
Enterococcus faecalis strain E512 plasmid pE512	100	0	100.00
Enterococcus faecalis 62 plasmid EF62pB	100	0	100.00
Enterococcus faecalis strain CVM N48037F plasmid pN48037F-2	100	0	99.92
Enterococcus faecalis strain sorialis plasmid Efsorialis-p2	100	0	99.92
